# Retraction Note: Open reduction and closed reduction internal fixation in treatment of femoral neck fractures: a meta-analysis

**DOI:** 10.1186/s12891-015-0528-z

**Published:** 2015-03-26

**Authors:** Weiguo Wang, Junjie Wei, Zhanwang Xu, Wenkun Zhuo, Yuan Zhang, Hui Rong, Xuecheng Cao, Pingshan Wang

**Affiliations:** Department of Orthopaedic Surgery, General Hospital of Jinan Military Command, 250031 Jinan, China; Research on 2013 stage doctoral student of TCM Orthopaedics, Shandong University of Traditional Chinese Medicine, Jinan, China; Outpatient Department, General Hospital of Jinan Military Command, 250031 Jinan, China; Department of Orthopaedics, The First Affiliated Hospital of Shandong University of Traditional Chinese Medicine, NO.16369, Jingshi Road, 250014 Jinan, China

## Retraction

The Publisher and Editor regretfully retract this article [1] because the peer-review process was inappropriately influenced and compromised. As a result, the scientific integrity of the article cannot be guaranteed. A systematic and detailed investigation suggests that a third party was involved in supplying fabricated details of potential peer reviewers for a large number of manuscripts submitted to different journals. In accordance with recommendations from COPE we have retracted all affected published articles, including this one. It was not possible to determine beyond doubt that the authors of this particular article were aware of any third party attempts to manipulate peer review of their manuscript.
